# Association of Plasma Protein Risk Score and Risk of Cognitive Impairment in the Framingham Heart Study

**DOI:** 10.1002/alz.091918

**Published:** 2025-01-09

**Authors:** Habbiburr Rehman, Ting Fang Alvin Ang, Qiushan Tao, Rhoda Au, Lindsay A. Farrer, Wei Qiao Qiu, Xiaoling Zhang

**Affiliations:** ^1^ Boston University School of Medicine, Boston, MA USA; ^2^ Department of Anatomy & Neurobiology, Boston University Chobanian & Avedisian School of Medicine, Boston, MA USA; ^3^ Boston University Chobanian & Avedisian School of Medicine, Boston, MA USA; ^4^ Department of Pharmacology, Physiology & Biophysics, Boston University Chobanian & Avedisian School of Medicine, Boston, MA USA; ^5^ The Framingham Heart Study, Boston University School of Medicine; Boston University School of Public Health, Boston, MA USA; ^6^ Alzheimer’s Disease Research Center, Boston University Chobanian & Avedisian School of Medicine, Boston, MA USA; ^7^ Department of Anatomy and Neurobiology, Neurology and Medicine, Framingham Heart Study, BU Alzheimer's Disease Research Center, Boston University Chobanian & Avedisian School of Medicine, Boston, MA USA; ^8^ Department of Medicine (Biomedical Genetics), Boston University Chobanian & Avedisian School of Medicine, Boston, MA USA; ^9^ Department of Neurology, Boston University Chobanian & Avedisian School of Medicine, Boston, MA USA; ^10^ Framingham Heart Study, Boston University Chobanian & Avedisian School of Medicine, Boston, MA USA; ^11^ Departments of Medicine (Biomedical Genetics), Neurology, Ophthalmology, Biostatistics, and Epidemiology, Boston University Schools of Medicine and Public Health, Boston, MA USA; ^12^ Department of Biostatistics, Boston University School of Public Health, Boston, MA USA; ^13^ Department of Psychiatry, Boston University Chobanian & Avedisian School of Medicine, Boston, MA USA; ^14^ Boston University School of Public Health, Boston, MA USA; ^15^ Boston University Department of Medicine (Biomedical Genetics), School of Medicine, Boston, MA USA

## Abstract

**Background:**

Plasma‐based biomarkers are emerging as a new diagnostic tool for Alzheimer’s disease (AD) with high accuracy and low cost. Whether aggregated plasma protein risk score (PPRS) derived from a single sample could be useful for diagnosis of incident mild cognitive impairment (MCI) and AD is uncertain. This study aims to understand the association between PPRS and future risk of AD related outcomes.

**Method:**

We included Framingham Heart Study (FHS) Offspring cohort participants with plasma aptamer based SOMAscan proteomics assay data at exam 5. After excluding subjects who were have missing data for AD risk factors (age, sex, education and APOE ɛ4) and had other‐types of dementia, 1523 participants (cognitive normal (CN)=1258, MCI=137, AD=128) were considered for the primary analyses. The Cox model with LASSO penalty was used to build the PPRs for AD and MCI. The association of PPRS for AD risk was tested for outcomes of MCI/AD incidence (mean follow‐up=23.06 years), brain volume, and blood AD biomarkers.

**Result:**

We identified 279 proteins for inclusion in the MCI PPRS and 103 proteins for inclusion in the AD PPRS. The MCI PPRS had a hazard ratio (HR) of 1.79 [4.99, 7.37] and the discriminating power (C‐index) was 0.86, and after including risk factors C‐index was 0.88. The AD PPRS had a HR of 1.79 [4.86,7.33] and the C‐index was 0.89 and after including the risk factors the power of the model was improved (C‐index=0.92). The chance of developing AD for a CN participant at age 80 years with in 20 years with high AD PPRS was 85% and with low AD PPRS was 15%. Brain volume distribution using 8 variables was significantly decreasing with increasing level of MCI PPRS (p‐value<0.001 for 5 variables) and AD PPRS (p‐value<0.001 for all 8 variables). The increased levels of MCI PPRS (p‐value<0.001) and AD PPRS (p‐value<0.001) were also significantly associated with increased plasma p‐tau181.

**Conclusion:**

This study suggests that aggregated protein risk scores from plasma samples can be used to predict future risk of MCI and AD, thus serving as a potential screening tool for early intervention to prevent AD or slow its progression.

